# Nanoengineering Solutions for Cancer Therapy: Bridging the Gap between Clinical Practice and Translational Research

**DOI:** 10.3390/jcm13123466

**Published:** 2024-06-13

**Authors:** Pankaj Garg, Siddhika Pareek, Prakash Kulkarni, Ravi Salgia, Sharad S. Singhal

**Affiliations:** 1 Department of Chemistry, GLA University, Mathura 281406, Uttar Pradesh, India; 2 Department of Medical Oncology and Therapeutics Research, Beckman Research Institute of City of Hope, Comprehensive Cancer Center and National Medical Center, Duarte, CA 91010, USA

**Keywords:** nanoengineering, clinical practice, translational research, preclinical models, targeted delivery

## Abstract

Nanoengineering has emerged as a progressive method in cancer treatment, offering precise and targeted delivery of therapeutic agents while concurrently reducing overall toxicity. This scholarly article delves into the innovative strategies and advancements in nanoengineering that bridge the gap between clinical practice and research in the field of cancer treatment. Various nanoengineered platforms such as nanoparticles, liposomes, and dendrimers are scrutinized for their capacity to encapsulate drugs, augment drug efficacy, and enhance pharmacokinetics. Moreover, the article investigates research breakthroughs that drive the progression and enhancement of nanoengineered remedies, encompassing the identification of biomarkers, establishment of preclinical models, and advancement of biomaterials, all of which are imperative for translating laboratory findings into practical medical interventions. Furthermore, the integration of nanotechnology with imaging modalities, which amplify cancer detection, treatment monitoring, and response assessment, is thoroughly examined. Finally, the obstacles and prospective directions in nanoengineering, including regulatory challenges and issues related to scalability, are examined. This underscores the significance of fostering collaboration among various entities in order to efficiently translate nanoengineered interventions into enhanced cancer therapies and patient management.

## 1. Introduction

Nanotechnology represents an innovative area with the potential to significantly revolutionize the treatment of cancer. When operating at the nanoscale, materials exhibit distinctive characteristics that facilitate precise handling and the directed conveyance of medications to cancerous cells. The primary objective of this manuscript is to investigate the impact of nanotechnology on the realm of cancer therapy, with a specific emphasis on its fundamental principles and utilization in contemporary oncology [1].

Conventional chemotherapy frequently encounters challenges such as inadequate drug solubility, restricted efficacy, and systemic side effects. These obstacles are effectively surmounted by nanotechnology through the encapsulation of drugs within nanocarriers, thus safeguarding them from degradation and enhancing their systemic distribution. Nanoparticles possess the capability to traverse biological barriers such as the blood-brain barrier, thereby introducing novel prospects for the treatment of previously intractable brain malignancies [2]. Tailorable nanoengineered drug conveyance systems, encompassing nanoparticles, liposomes, and dendrimers, can be designed to accumulate in tumors either through passive means (leveraging the enhanced permeability and retention effect) or actively (via the attachment of specific molecules or antibodies). This targeted strategy serves to diminish side effects while amplifying the efficacy of cancer therapies [3].

Nanotechnology’s application in cancer treatment is distinguished by its ability to amalgamate therapy and diagnosis within a singular platform, referred to as theranostics [4]. The utilization of theranostic nanoparticles facilitates both tumor imaging and precise delivery of treatments. This holistic approach allows for real-time assessment of treatment efficacy, early detection of cancer recurrence, and the tailoring of treatment strategies based on individual patient attributes [5]. Within the field of oncology, nanotechnology assumes a pivotal role in propelling precision medicine through molecular targeting methodologies. Customized therapeutic interventions guided by biomarkers specific to the molecular profile of each tumor enhance treatment outcomes while mitigating adverse effects [6]. Moreover, the integration of nanosensors and nanodevices amplifies the sensitivity of cancer biomarker identification, thereby assisting in early diagnosis and prognosis [7]. Nanotechnology represents a substantial transformation in cancer therapy, providing exact, targeted, and personalized treatment modalities. By bridging the disconnect between laboratory advancements and clinical application, nanotechnology holds the promise of ameliorating patient outcomes, diminishing treatment side effects, and ushering in a novel epoch in cancer management [8].

## 2. Nanoengineering Approaches for Targeted Drug Delivery

Nanoengineering has revolutionized the administration of medications in cancer therapy by facilitating accurate and targeted distribution of therapeutic substances to tumor sites while minimizing overall toxicity. This section delves into various nanoengineering techniques utilized for targeted drug delivery, emphasizing their mechanisms, advantages, and obstacles [9].

### 2.1. Nanoparticles as Vehicles for Medications

Nanoparticles function as adaptable carriers for medications with distinctive attributes such as a substantial surface area-to-volume ratio, customizable dimensions and structure, and modifiable surface chemistry [10]. They have the capacity to encase diverse therapeutic substances, ranging from chemotherapeutic medications to nucleic acids, peptides, and proteins. Nanoparticles shield medications from deterioration, prolong their circulation period, and govern their release patterns. Nanoparticles can be engineered to target specific tissues or cells, enhancing the precision of drug delivery, and minimizing side effects. This targeted delivery is achieved through surface modifications that enable the nanoparticles to recognize and bind to specific biomarkers on diseased cells. Additionally, the ability to penetrate biological barriers, such as the blood-brain barrier, opens new avenues for treating central nervous system disorders [11].

### 2.2. Liposomes for Enclosing Hydrophobic and Hydrophilic Medications

Liposomes, lipid-based nanocarriers resembling cellular membranes, can enclose both hydrophobic and hydrophilic medications [12]. Their dual-layered structure permits the integration of lipophilic medications in the lipid bilayer and hydrophilic medications in the aqueous core. Liposomes enhance medication solubility, accessibility, and target precision, rendering them efficient carriers for chemotherapy and targeted treatments. Liposomes exhibit biocompatibility and biodegradability, reducing the risk of adverse reactions and making them suitable for clinical applications. Their ability to fuse with cellular membranes facilitates the direct delivery of encapsulated drugs into the cytoplasm, enhancing therapeutic efficacy. The surface of liposomes can be modified with polyethylene glycol (PEG) to create “stealth” liposomes, which evade detection and clearance by the immune system, thereby extending the circulation time of the drug [13]. This extended circulation allows for increased accumulation of the drug in targeted tissues, particularly in tumors, through the enhanced permeability and retention (EPR) effect.

### 2.3. Polymeric Nanoparticles for Regulated Release

Polymeric nanoparticles like poly (lactic-co-glycolic acid) (PLGA) and polyethylene glycol (PEG)-based polymers are frequently employed for controlled medication release [14]. These nanoparticles can be tailored to steadily release medications, extending their therapeutic impacts, and reducing dosing frequency. Surface modifications with targeting ligands enhance their specificity to cancer cells while mitigating off-target effects. Polymeric nanoparticles offer enhanced safety as they biodegrade without harmful residues. They are versatile, encapsulating various therapeutic agents for multifunctional drug delivery. Advancements include stimuli-responsive types that release drugs in response to body triggers, optimizing efficacy and reducing side effects. These nanoparticles can penetrate biological barriers, like the blood-brain barrier, enabling treatment in previously inaccessible areas, expanding therapeutic possibilities [15].

### 2.4. Dendrimers for Accurate Medication Delivery

Dendrimers, complex tree-like nanostructures with precise molecular weights and sizes, provide controlled medication loading and release kinetics, making them suitable for targeted drug delivery. They can be personalized with targeting agents, imaging substances, and therapeutic payloads, enabling diverse strategies for cancer detection and therapy. Their highly branched architecture provides numerous surface functional groups, allowing for the attachment of various functional moieties, such as drugs, targeting ligands, and imaging agents. This multi-functionality enhances the specificity and efficacy of dendrimer-based therapies [16]. Moreover, dendrimers exhibit low toxicity and high biocompatibility, making them safe for biomedical applications. Their nanoscale size allows for efficient penetration into tissues and cells, facilitating the delivery of therapeutic agents to the intended site of action. Additionally, dendrimers can be designed to respond to specific physiological conditions, such as pH or redox potential, enabling controlled and targeted release of their payload [16].

### 2.5. Nanoparticle Surface Functionalization for Targeting

The functionalization of nanoparticle surfaces is critical for targeted drug delivery. Through the attachment of specific functional groups like antibodies, peptides, aptamers, or small molecules onto nanoparticle surfaces, they can identify and bind to receptors or biomarkers overexpressed on cancer cells. This active targeting enhances drug accumulation at tumor sites while minimizing exposure to healthy tissues. Furthermore, surface functionalization can improve the pharmacokinetics and bio-distribution of nanoparticles, extending their circulation time and increasing their stability in the bloodstream [17]. This leads to more efficient delivery of therapeutic agents to the desired site. Functionalized nanoparticles can evade the immune system, reducing clearance by macrophages and enhancing their ability to reach tumor cells. Advancements in surface engineering also allow for the creation of multi-functional nanoparticles capable of delivering multiple therapeutic agents simultaneously. This can lead to synergistic effects, enhancing the overall therapeutic outcome. The integration of imaging agents into the surface-functionalized nanoparticles further facilitates real-time monitoring of drug delivery and treatment efficacy [17].

### 2.6. Stimuli-Responsive Nanoparticles for Induced Drug Release

Stimuli-responsive nanoparticles are engineered to release medications in reaction to environmental cues in the tumor microenvironment, such as pH, temperature, enzymes, or light [18]. This mechanism of induced drug release enhances drug delivery efficacy, diminishes premature release in non-targeted tissues, improves therapeutic outcomes, and reduces side effects. Stimuli-responsive nanoparticles can be designed to undergo structural changes, such as swelling or degradation, in response to the targeted indicators, thereby releasing the encapsulated drugs in a controlled manner. This adaptability ensures that the therapeutic agents are released at the optimal time and location, maximizing their efficacy while minimizing harm to healthy cells. Recent developments in this field include nanoparticles responsive to more sophisticated stimuli, such as magnetic fields or ultrasound, offering non-invasive methods to trigger drug release remotely [19]. This advanced control over drug delivery holds great promise for enhancing the treatment of complex diseases, including cancer.

## 3. Nano-Based Imaging Techniques for Cancer Detection and Monitoring

**Figure 1:**
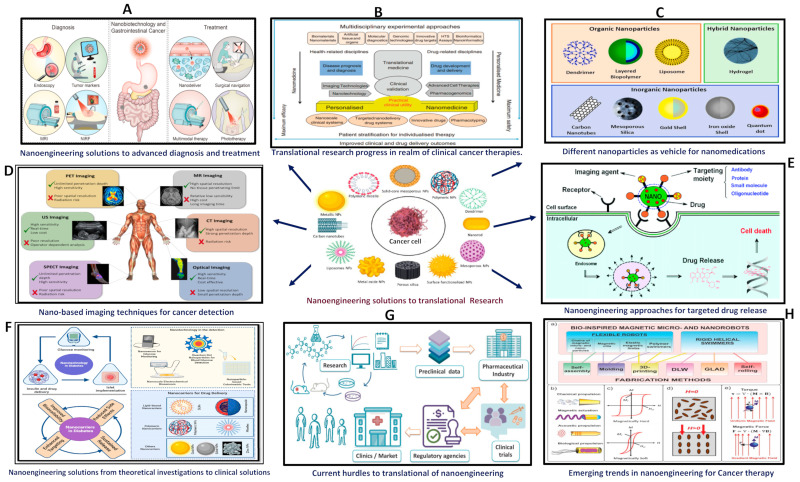
Schematic overview of advanced nanotechnology applications. (**A**) Innovations in nano-engineering for cutting-edge diagnosis and therapeutic solutions. (**B**) The pivotal role of translational research in advancing clinical therapies and addressing challenges. (**C**) Diverse nanoparticles and their applications illustrated schematically. (**D**) Various nano-based advanced molecular imaging modalities depicted. (**E**) Mechanism of action for functionalized nanoparticles delivering anticancer drugs with targeted ligands. (**F**) Nanoparticles as sophisticated drug delivery systems and controlled release carriers. (**G**) Challenges in nano-based translational research, including regulatory and ethical considerations. (**H**) Detailed subpanels on bioinspired magnetic micro and nanorobots: (**a**) Fabrication methods for magnetic micro and nano-robots for cellular-level surgical cancer treatments using hyperthermia, (**b**) Actuation techniques for biomimetic micro and nano-robots, (**c**) Magnetization dependence on external magnetic fields, (**d**) Polymer curing with magnetic nanoparticles in the presence and absence of a magnetic field, and (**e**) Movement dynamics of magnetic nanoparticles in uniform and non-uniform magnetic fields.

Nano-based imaging methodologies have revolutionized the field of cancer detection and monitoring through the provision of elevated sensitivity, specificity, and real-time visualization of tumors at both the molecular and cellular levels [20]. This section explores the diverse nano-based imaging approaches utilized in oncology, analyzing their mechanisms, applications, advantages, and obstacles (Figure 1; Table 1 and Table 2).

### 3.1. Fluorescence Imaging with Quantum Dots

Quantum dots (QDs) are minuscule semiconductor crystals that emit fluorescence upon excitation, showcasing remarkable optical characteristics such as brightness, stability, and precise emission spectra, rendering them well-suited for fluorescence imaging [34]. Through the attachment of targeting ligands, QDs can selectively adhere to cancer cells or biomarkers, facilitating accurate tumor visualization. The utilization of multiplexed imaging with QDs enables the simultaneous observation of numerous molecular targets, thereby supporting comprehensive diagnostics and the development of personalized treatment strategies. Recent advancements in QD technology have also focused on developing biocompatible and environmentally friendly QDs to address concerns regarding toxicity and environmental impact. These efforts aim to broaden the clinical application of QD-based fluorescence imaging in cancer research and patient care. Additionally, the integration of QDs with other imaging modalities, such as magnetic resonance imaging (MRI) or computed tomography (CT), offers multimodal imaging capabilities that combine the strengths of each technique, providing complementary information for more accurate and comprehensive cancer diagnosis and treatment planning [35,36].

### 3.2. Magnetic Resonance Imaging (MRI) with Iron Oxide Nanoparticles

Iron oxide nanoparticles act as MRI contrast agents due to their magnetic properties and compatibility with biological systems. These nanoparticles possess the ability to accumulate in tumor tissues, heightening contrast on MRI scans and enabling non-invasive visualization of tumor characteristics [36]. Tailored iron oxide nanoparticles with targeting moieties enhance MRI contrast specifically for tumors, aiding in early identification, precise staging, and assessment of treatment responses in individuals with cancer. Iron oxide nanoparticles can further be functionalized with ligands, antibodies, or peptides to target specific tumor markers, thereby increasing the specificity and sensitivity of MRI diagnostics [37]. This targeted approach not only improves the accuracy of tumor detection but also allows for the differentiation between malignant and benign tissues, facilitating more informed clinical decision-making. The biocompatibility and biodegradability of iron oxide nanoparticles make them safe for repeated imaging sessions, providing a valuable tool for monitoring the progression of disease and the effectiveness of therapeutic interventions over time. Furthermore, their potential for integration with therapeutic agents opens the door to theranostic applications, where diagnosis and treatment can be combined into a single, efficient process [37].

### 3.3. Gold Nanoparticles for Surface-Enhanced Raman Scattering Imaging

Gold nanoparticles exhibit distinctive optical features such as localized surface plasmon resonance (LSPR), which amplify Raman scattering signals emitted by neighboring molecules [31]. This phenomenon, referred to as surface-enhanced Raman scattering, facilitates highly sensitive molecular imaging of cancer biomarkers present within tumors. Functionalized gold nanoparticles have the capability to target particular molecules on cancerous cells, enabling precise SERS imaging and molecular characterization for diagnostic purposes and monitoring of therapeutic outcomes. Gold nanoparticles can be engineered to carry therapeutic payloads alongside their imaging capabilities, creating theranostic agents that combine diagnosis and treatment in a single platform. This approach allows for real-time monitoring of treatment response while delivering targeted therapy directly to cancer cells, optimizing therapeutic efficacy [38]. Moreover, the unique properties of gold nanoparticles, such as their biocompatibility and stability, make them suitable for clinical applications in cancer imaging and therapy. Their tunable size and shape further enhance their performance as surface-enhanced Raman scattering imaging agents, providing researchers and clinicians with valuable insights into the molecular landscape of tumors.

### 3.4. Photoacoustic Imaging with Nanoparticles

Photoacoustic imaging merges laser-induced signals with ultrasound detection to visualize tissue structures and molecular properties. Nanoparticles like carbon nanotubes or organic dyes function as contrast agents by absorbing laser energy and generating acoustic waves [30]. This imaging modality offers significant tissue penetration depth, superior resolution, and functional insights into tumor attributes such as vasculature, oxygenation levels, and molecular targets. Nanoengineered photoacoustic probes present promising prospects for non-invasive visualization of cancer and guided therapeutic interventions. The versatility of nanoparticle-based contrast agents allows for the design of multifunctional probes that can simultaneously target multiple biomarkers or cellular processes within tumors. This multifaceted approach enhances the specificity and accuracy of photoacoustic imaging, providing a comprehensive view of tumor biology and aiding in treatment decision-making [39]. Additionally, advancements in nanoparticle engineering enable the development of photoacoustic probes with controlled release properties. These probes can deliver therapeutic agents in a spatiotemporally controlled manner, precisely targeting tumor sites while minimizing systemic exposure and off-target effects.

## 4. Emerging Trends in Nanoengineering for Cancer Therapy

The utilization of nanoengineering in cancer therapy has rapidly emerged as a promising approach, providing precise and targeted solutions to combat the complexities of this disease. A variety of novel trends in nanoengineering are reshaping the landscape of cancer therapy, transforming treatment strategies, enhancing patient outcomes, and paving the way for personalized medicine [40]. Here are detailed insights into some of the key emerging trends in nanoengineering for cancer therapy.

### 4.1. Personalized Nanomedicine

The concept of personalized medicine is increasingly prevalent in the realm of cancer treatment, with nanoengineering assuming a crucial role. Nanoparticles have the capacity to be customized for the delivery of specific medications, targeting molecules, or therapeutic agents tailored to the unique profiles of individual patients, the characteristics of tumors, and genetic variances. This personalized approach to nanomedicine heightens the effectiveness of treatment, diminishes adverse effects, and enhances patient responses. Advanced diagnostic techniques like genomic analysis and imaging enable precise identification of molecular targets, guiding nanoparticle design for optimal therapeutic outcomes tailored to patients. Personalized nanomedicine incorporates patient-specific data, improving efficacy, reducing resistance, and optimizing outcomes through ongoing monitoring and adjustments. Ongoing research in nanomedicine expands possibilities, promising revolutionary, highly targeted therapies that maximize benefits and minimize risks in cancer care [21].

### 4.2. Multifunctional Nanoparticles

Multifunctional nanoparticles are crafted to encompass a multitude of functions within a single platform, offering versatile solutions for cancer therapy [22]. These nanoparticles can integrate functions such as drug delivery, imaging capabilities, targeting ligands, and therapeutic payloads, facilitating both diagnosis and therapy (known as theranostics). Through the amalgamation of diverse functions, multifunctional nanoparticles refine treatment accuracy, enable real-time monitoring, and support individualized treatment strategies. Multifunctional nanoparticles can be designed to release therapeutic agents in response to tumor-specific stimuli, minimizing damage to healthy tissues and enhancing cancer treatment safety and efficacy. They also integrate imaging agents for real-time visualization of drug distribution and tumor response, aiding in treatment adjustment. These nanoparticles can deliver combination therapies to enhance outcomes and overcome drug resistance [23]. Advances in this field are expanding their functionalities to include gene delivery and immune modulation, paving the way for personalized oncology.

### 4.3. Stimuli-Responsive Nanomaterials

Stimuli-responsive nanomaterials are engineered to react to specific signals within the tumor microenvironment, such as pH levels, temperature variations, enzymatic activity, or light exposure. These materials alter or release drugs upon encountering stimuli, resulting in controlled drug release at sites of tumors. Stimuli-responsive nanomaterials heighten the efficacy of treatment, decrease off-target effects, and combat drug resistance, presenting innovative options for tailored and adaptive therapies [24]. The dynamic nature of stimuli-responsive nanomaterials allows for fine-tuning drug release kinetics, optimizing therapeutic outcomes while minimizing systemic toxicity. These materials can also be designed to respond selectively to tumor-specific stimuli, enhancing targeting precision and reducing impact on healthy tissues. The versatility of stimuli-responsive nanomaterials extends beyond drug delivery, with applications in diagnostic imaging, therapy monitoring, and therapeutic agent activation within tumors. The integration of stimuli-responsive properties with multifunctional nanoplatforms enables multifaceted approaches to cancer treatment, enhancing treatment efficacy and patient outcomes [24].

### 4.4. Targeting the Tumor Microenvironment

Nanoengineering approaches are focused on targeting elements of the tumor microenvironment that are pivotal for cancer advancement, metastasis, and resistance to treatment. Targeted nanotherapeutics strive to modify the tumor microenvironment, fostering an inhospitable milieu for tumor proliferation, enhancing immune reactions, and amplifying treatment results [25]. Nanoengineered treatments target specific components of the tumor microenvironment, disrupting processes crucial for tumor growth and sensitizing cancer cells to conventional therapies, improving outcomes. They also deliver immunomodulatory agents to activate anti-tumor immune responses synergistically with traditional treatments, leading to enhanced tumor regression. Smart nanoparticles respond to tumor microenvironment changes, optimizing drug release and minimizing off-target effects, promising a revolution in cancer treatment paradigms [25].

### 4.5. Nanotechnology in Immunotherapy

Nanotechnology is enhancing immunotherapy by augmenting the delivery of drugs to immune cells, bolstering the activation of immune cells, and circumventing immunosuppressive mechanisms within the tumor microenvironment. Nanoparticles can transport immunomodulatory agents, checkpoint inhibitors, cytokines, vaccines, or antigen-presenting molecules to immune cells or tumor sites, intensifying anti-tumor immune responses and yielding enduring treatment effects [27]. Nanotechnology allows for the engineering of nanoparticles with specific properties that enhance their interactions with immune cells. Surface modifications can promote targeted binding to immune receptors, enhancing uptake and activation of immune cells against cancer cells. Additionally, nanoscale delivery systems can protect fragile immunotherapeutic agents from degradation in the bloodstream, ensuring their integrity and potency until they reach their intended targets. This protection prolongs the therapeutic effects and reduces the required dosage, minimizing potential side effects. Moreover, nanotechnology enables the development of personalized immunotherapy approaches by tailoring nanoparticle formulations to individual patient profiles. This customization enhances treatment efficacy and reduces the risk of adverse reactions, ultimately improving patient outcomes [33].

### 4.6. Bioinspired Nanomaterials

Bioinspired nanomaterials mimic biological structures and processes to heighten the delivery of drugs and therapeutic efficacy. Instances include biomimetic nanoparticles, liposomes, and exosome-based systems for drug delivery. These materials enhance biocompatibility, cellular uptake, and targeted delivery, contributing to safer and more efficient cancer treatments [41]. Furthermore, these materials can be engineered to replicate the surface proteins and lipids of cell membranes, enhancing their ability to fuse with target cells and deliver their therapeutic payload directly into the cytoplasm. This biomimetic approach not only increases the precision of drug delivery but also reduces the likelihood of off-target effects, thereby improving the overall safety profile of the treatment [41].

### 4.7. Nanotechnology in Combination Therapies

The realm of nanoengineering facilitates the development of combination therapies, where multiple therapeutic agents or approaches are amalgamated within a single nanoplatform [26]. Combination therapies concurrently target distinct pathways within tumors, surmount drug resistance, and enhance treatment outcomes. Nanotechnology enables the controlled release of multiple agents, improves the synergy of drugs, and decreases systemic toxicity, rendering combination therapies a promising tactic in cancer treatment [42]. Nanoengineered combination therapies influence tumor-specific characteristics to optimize treatment response and personalize patient care. The versatility of nanoplatforms enables the integration of various therapeutic modalities, from chemotherapy to gene therapy, creating tailored treatment regimens. Incorporating imaging components allows real-time monitoring, enhancing precision and reducing resistance, fostering adaptive treatment strategies. Ongoing nanoengineering research explores innovative strategies like integrating nanoparticles with physical stimuli, promising improved efficacy and overcoming treatment challenges in cancer management [43].

In conclusion, the evolving trends in nanoengineering for cancer therapy exemplify the ongoing progression and creativity within the discipline. Through the utilization of nanotechnology, scientists and healthcare professionals are able to tackle significant obstacles in the realm of cancer management, administer individualized and precise treatments, surmount treatment resilience, and ultimately enhance patient results in the battle against cancer.

## 5. The importance of Translational Research Progress in the Realm of Clinical Cancer Therapies

Translational research is imperative in bridging fundamental scientific discoveries with practical clinical applications, particularly in the field of cancer treatment (Figure 1; Table 1 and Table 2). This section delves into significant insights and recent advancements in translational research, emphasizing the conversion of scientific findings into innovative cancer treatments and technologies [44].

### 5.1. Identification and Validation of Biomarkers

The initiation of translational research frequently involves the discovery and validation of biomarkers that can predict disease advancement, response to treatment, and patient outcomes [28]. Progress in genomics, proteomics, and other omics technologies has facilitated the identification of novel biomarkers associated with cancer progression, drug resistance, and metastasis. The transition of these biomarkers from laboratory settings to clinical application requires thorough validation studies utilizing patient samples, leading to their incorporation into diagnostic assays, patient stratification methodologies, and targeted therapeutic approaches [45]. Integration of AI and machine learning boosts efficiency and accuracy in identifying clinically relevant biomarkers, uncovering subtle patterns for translation into clinical practice. Collaborations among researchers, clinicians, and industry partners are pivotal in validating and implementing biomarkers, fostering innovation and scalability in diagnostic and therapeutic strategies. Advancements in biomarker technologies like liquid biopsies and sequencing platforms offer non-invasive, real-time monitoring of disease and treatment responses, enhancing personalized cancer management [45].

### 5.2. Utilization of Preclinical Models for Drug Development

Preclinical models, including cell lines, organoids, genetically engineered mouse models, and patient-derived xenografts, are indispensable for evaluating the efficacy and safety of potential cancer therapies prior to clinical trials. Translational researchers leverage these models to explore tumor biology, assess candidate drugs, investigate resistance mechanisms, and pinpoint treatment targets. Recent advancements in 3D culture systems and organ-on-a-chip technologies have heightened the relevance and predictive capacity of preclinical models, expediting the development of novel cancer treatments [29]. Integration of computational modeling and simulation with preclinical models enhances predictive power and reduces drug development time/cost. Virtual testing simulates drug interactions, predicts responses, and optimizes dosing, boosting drug candidate success rates. Diverse preclinical models like patient-derived organoids promote precision medicine by mimicking patient-specific tumor characteristics, enabling personalized treatment strategies and clinical success. Collaborations among academia, industry, and regulators standardize model use, validate relevance, and ensure ethical practices, translating preclinical findings into impactful clinical outcomes, benefiting global cancer patients [32].

### 5.3. Development of Biomaterials and Drug Delivery Systems

Innovative biomaterials and drug delivery systems generated through translational research have revolutionized cancer therapy. Nanoparticles, liposomes, hydrogels, and implantable devices are engineered to transport therapeutic agents directly to tumor sites, enhancing drug efficacy, diminishing side effects, and overcoming drug resistance [46]. Translational endeavors concentrate on refining drug delivery systems for specific cancer types, improving pharmacokinetics, and integrating imaging and therapeutic functionalities into versatile platforms [47]. Advancements in biomaterials design focus on enhancing biocompatibility, stability, and controlled release properties, ensuring targeted and sustained drug delivery while minimizing systemic toxicity. These developments enable the precise modulation of drug release kinetics, allowing for tailored dosing regimens and optimized therapeutic outcomes. Additionally, the incorporation of stimuli-responsive elements in drug delivery systems allows for triggered drug release in response to specific cues within the tumor microenvironment, further enhancing treatment efficacy and reducing off-target effects. This dynamic approach to drug delivery ensures that therapeutic agents are delivered precisely when and where they are needed, maximizing their impact on cancer cells while sparing healthy tissues [47].

### 5.4. Progress in Immunotherapy and Precision Medicine

Translational research has been instrumental in advancing immunotherapy and precision medicine strategies for treating cancer [48]. Immune checkpoint inhibitors, adoptive cell therapies, cancer vaccines, and chimeric antigen receptor (CAR) T-cell therapies have emerged as potent immunotherapeutic approaches, resulting in durable responses and improved survival rates for certain cancers. Precision medicine initiatives utilize molecular profiling, biomarker-guided treatments, and personalized therapeutic plans to provide individualized care and enhance therapeutic outcomes [49].

### 5.5. Execution of Clinical Trials and Negotiation of Regulatory Pathways

Translational researchers collaborate with clinicians and regulatory authorities to develop and implement clinical trials that evaluate the safety, effectiveness, and tolerability of new cancer treatments. These trials encompass phase I-III investigations, biomarker-oriented trials, combination therapy studies, and innovative trial structures (such as basket and umbrella trials) to concurrently assess multiple therapies [50]. Insights from preclinical research inform the design of clinical trials, criteria for patient selection, dosage adjustments, and strategies for monitoring treatment, facilitating the successful translation of promising therapies into clinical settings [51].

## 6. Challenges Associated with Progressing Cancer Therapies via Translational Research

The translation of scientific discoveries into practical applications benefiting patients is crucial in advancing cancer treatments through translational research. Despite notable progress, challenges such as limited funding, data reproducibility issues, patient diversity, and regulatory complexities hinder translational research [52]. Moving forward, efforts may focus on fostering collaboration among academia, industry, and healthcare institutions, integrating artificial intelligence and big data analytics into translational processes, advancing organoid and patient-derived models for personalized medicine, and addressing disparities in accessing innovative therapies. The sustained investment in translational research infrastructure, training programs, and interdisciplinary collaborations is pivotal for driving significant advancements in cancer care and improving patient outcomes [53].

### 6.1. Complexity of Tumor Biology

Cancer, being a complex disease with multiple subtypes and molecular mechanisms, poses challenges in developing effective targeted therapies across various cancer types and stages due to tumor heterogeneity and interactions with the microenvironment. Future perspectives entail integrating multi-omics data, utilizing artificial intelligence for data analysis, and exploring combination therapies to address tumor diversity [54]. The advent of CRISPR-based genome editing technologies has opened new avenues for developing personalized therapies by precisely targeting genetic alterations driving tumor growth. CRISPR-based approaches, combined with advances in gene therapy and immunotherapy, hold promise for tailored interventions that can effectively modulate tumor biology and enhance treatment responses [54].

### 6.2. Development of Drug Resistance

The emergence of drug resistance in cancer cells leading to treatment failure and disease progression necessitates the identification of resistance mechanisms and the development of strategies to overcome them through translational research, such as combination therapies, drug repurposing, and adaptive treatment approaches based on real-time tumor response monitoring [55]. Future outlooks involve identifying predictive resistance biomarkers and devising personalized treatment plans. Future strategies to combat drug resistance in cancer involve identifying predictive biomarkers linked to resistance mechanisms using advanced technologies like genomics, proteomics, and imaging [55]. Understanding genetic mutations, protein changes, or phenotypic shifts that cause resistance allows for personalized treatment plans targeting specific mechanisms, optimizing outcomes for each patient.

### 6.3. Design and Implementation of Clinical Trials

The meticulous planning, patient selection, and regulatory approvals are imperative for the design and execution of clinical trials for novel cancer therapies [56]. Translational researchers face challenges in patient recruitment, endpoint selection, and data interpretation. Future perspectives include innovative trial designs like basket and umbrella trials, biomarker-driven approaches, and the integration of real-world evidence to streamline trials and accelerate therapy approvals. Integration of real-world evidence (RWE) in clinical trials enhances understanding of treatment outcomes, patient preferences, and healthcare utilization outside controlled settings. RWE supplements traditional trial data, offering broader insights into treatment efficacy, safety, and long-term impacts, aiding in decision-making and therapy approvals [57]. Advancements in digital health tech like EHRs, telemedicine, and wearables improve remote monitoring, data accuracy, and patient engagement, enhancing trial efficiency. Collaborative efforts among stakeholders, including researchers, clinicians, regulators, industry, and patient advocates, are crucial for overcoming trial challenges and accelerating transformative cancer therapies, benefiting patients globally.

### 6.4. Regulatory and Ethical Considerations

Navigating regulatory pathways, ensuring patient safety, and addressing ethical considerations are critical aspects of translational research. Regulatory authorities necessitate robust preclinical and clinical data for therapy approvals, which can be resource-intensive and time-consuming [58]. Future perspectives involve fostering collaboration among researchers, clinicians, regulatory bodies, and industry partners to expedite therapy development while upholding safety and ethical standards. Efforts to enhance transparency, data sharing, and regulatory harmonization are essential for expediting the translation of promising therapies from bench to bedside. Moreover, initiatives promoting patient-centered approaches, informed consent, and ethical guidelines play a vital role in ensuring patient safety and maintaining public trust in biomedical research. Collaborative endeavors and ethical frameworks are indispensable in navigating the complex landscape of translational research and advancing innovative therapies for improved patient outcomes [59].

### 6.5. Equity and Accessibility

The challenge of accessing innovative cancer therapies persists, particularly in low-resource settings and underserved populations. Translational research must address healthcare access inequities, affordability issues, and knowledge gaps to ensure equitable distribution of life-saving treatments. Future perspectives include advocating for universal healthcare coverage, facilitating technology transfer to resource-constrained regions, and implementing educational and outreach initiatives [60].

Translational research holds significant promise in advancing cancer therapies; addressing challenges and embracing future perspectives are essential for realizing its full potential in enhancing patient outcomes, reducing the cancer burden, and ultimately achieving cancer control. Collaborative efforts, innovative strategies, and a patient-centered approach are crucial for overcoming these hurdles and driving transformative changes in cancer care [61].

## 7. Conclusions and Future Prospective

Nanoengineering has exerted a significant influence on the enhancement of cancer therapy, revolutionizing the domain of oncology through the provision of precise, targeted, and individualized remedies that hold the potential to significantly aid patients. By leveraging sophisticated nanomaterials such as nanoparticles, liposomes, and dendrimers, researchers and healthcare practitioners are able to accurately administer treatments to cancerous regions while minimizing the impact on healthy tissues [62]. The amalgamation of nanotechnology with imaging modalities facilitates the real-time monitoring of interventions and the early identification of ailments. Emerging patterns like personalized nanomedicine, adaptable nanoparticles, reactive nanomaterials, and targeted strategies tailored to the tumor microenvironment are reshaping cancer treatment, presenting auspicious avenues for more efficient and personalized therapeutic interventions [63].

The trajectory of cancer management is contingent upon the optimization of nanoengineering, as it grapples with challenges such as drug resistance, regulatory impediments, and scalability concerns. Collaborative endeavors involving scientists, clinicians, regulatory entities, and industry collaborators play a pivotal role in the translation of Nanoengineered solutions from theoretical investigations to practical applications in clinical settings. By addressing these hurdles and embracing the progressions in nanoengineering, we can inaugurate a novel epoch in cancer management characterized by enhanced survival rates, diminished adverse effects, and improved quality of life for cancer patients on a global scale [64].

## 8. Clinical Significance

Nanoengineering solutions play a crucial role in cancer treatment as they help connect scientific research with actual patient care. These solutions use advanced materials and methods to improve how to target tumors, deliver drugs, and deal with drug resistance. They also enable personalized treatments and better imaging and monitoring of the disease. Overall, nanoengineering has the potential to transform cancer therapy by making treatments more precise, overcoming obstacles like drug resistance, and ultimately improving patient outcomes and quality of life.

In summary, nanoengineering solutions for cancer therapy bridge the gap between laboratory discoveries and clinical applications by utilizing innovative nanomaterials and techniques to deliver precise and targeted treatments, ultimately improving patient outcomes while minimizing side effects and toxicity.

## Figures and Tables

**Table 1:** Structural overview of how nanoengineering solutions impact various aspects of cancer therapy, from targeted drug delivery and personalized medicine to enhanced immunotherapy and combination therapies.

Aspect of Nanoengineering	Description	Impact on Cancer Therapy	Reference Cited
**Targeted Drug Delivery**	Nanostructured systems such as nanoparticles, liposomes, and dendrimers facilitate the accurate administration of therapeutic substances to tumor locations, thus reducing overall systemic toxicity.	Improves the effectiveness of treatment, diminishes adverse outcomes, and circumvents resistance to medication.	[10]
**Personalized Nanomedicine**	Utilizing translational research findings, personalized treatments are customized based on individual patient profiles, biomarkers, and tumor characteristics.	Enhances treatment outcomes, increases patient response rates, and promotes personalized medicine strategies.	[21]
**Multifunctional Nanoparticles**	The integration of drug delivery, imaging capabilities, targeting ligands, and therapeutic payloads into a unified platform facilitates theranostic applications.	Enables the process of continuous monitoring, timely identification, and tailored therapeutic protocols.	[22,23]
**Stimuli-Responsive Nanomaterials**	Designed with the capability to react to specific signals in the tumor microenvironment, leading to controlled drug release and improved treatment specificity.	Minimizing off-target effects, overcoming drug resistance, and optimizing therapeutic efficacy are crucial objectives in pharmacology.	[24]
**Targeting Tumor Microenvironment**	Strategies in nanoengineering are centered on regulating elements of the tumor microenvironment to create an inhospitable condition for tumor proliferation and to stimulate immune reactions.	Improves the effectiveness of immunotherapy, hinders the advancement of tumors, and averts the spread of metastases.	[25]
**Combination Therapies**	Facilitating the creation of combination therapies, nanoengineered platforms incorporate various therapeutic approaches, synergistically targeting diverse pathways within tumors.	Enhances the synergy of treatment, surmounts mechanisms of resistance, and augments therapeutic results.	[26]
**Immunotherapy Enhancement**	Enhancements in immunotherapy are achieved through nanoengineered methods that optimize drug delivery to immune cells, stimulate immune responses, and overcome suppressive mechanisms in the tumor microenvironment.	Enhances the immune responses against tumors, extends the duration of responses to treatment, and diminishes the toxicity associated with immunotherapy.	[27]

**Table 2:** Clinical impact and patient outcomes in the context of the role of translational research for nanoengineering solutions of cancer therapy.

Nanoengineering Solution	Translational Research Contribution	Clinical Impact	Key Clinical Findings and Patient Outcomes	Reference Cited
**Nanoparticle Drug Delivery**	Generation of nanocarriers and kinetics tuning for medication release.	Improved medication efficiency and focused administration.	longer lifetimes, less systemic toxicity, and increased tumor response rates	[14]
	Preclinical research on pharmacokinetics and biodistribution		improved treatment tolerability and elevated therapeutic index	[15]
**Nanomedicine**	Finding biomarkers unique to tumors and optimizing nanomaterial design	Precision targeting of tumors and diseased cells	Reduced off-target effects and customized treatment methods.	[28]
	Clinical studies on the tolerance, safety, and effectiveness of nanomedicines		Enhanced disease management and extended progression-free survival	[29]
**Nanoparticle-based Imaging**	Creation of contrast agents and surface changes for nanoparticles	Improved diagnostic accuracy and imaging quality	Early tumor diagnosis, correct staging, well-planned therapy,	[30]
	Clinical verification of medicines based on nanoparticles for imaging modalities		Better treatment monitoring, and enhanced tumor margin visibility	[31]
**Nanotechnology in Radiotherapy**	Radiosensitization via nanoparticles, focusing on hypoxic areas in cancers	Enhanced radiation delivery and efficacy	Increased local tumor control, reduced radiation-related toxicity	[11]
	Preclinical research on tumor radiosensitivity and radiation dose enhancement		Improved tumor response, potential for dose escalation	[32]
**Theranostic Nanoparticles**	Imaging and therapeutic agents incorporated into a single nanoparticle	Simultaneous diagnosis and therapy	Personalized therapy modifications and real-time treatment response monitoring	[5]
	Theranostic agent clinical trials and patient-specific therapy planning		Better therapeutic results, less side effects, and shorter treatment times	[4]
**Nanoparticle-based Immunotherapy**	Nanoformulations of immunomodulatory agents, targeted immune cell delivery	Enhanced immune activation and response	Enhanced reaction times, long-lasting immunological recall, and decreased systemic adverse effects	[19]
	Clinical research on immune cell interactions and the effectiveness of immunotherapy		Increased tumor regression, extended survival, and possibility for long-term disease management	[33]

## Data Availability

No new data was created or analyzed in this study. Data sharing is not applicable to this article.
